# *Chlamydia muridarum* TC0066 is required for invasion and intracellular development in tissue culture but dispensable in the murine infection model

**DOI:** 10.1128/spectrum.02359-26

**Published:** 2026-09-30

**Authors:** Phuhai Nguyen, Konstantin V. Korotkov, Katerina Wolf, Kenneth A. Fields

**Affiliations:** 1 Department of Microbiology and Immunology & Molecular Genetics, University of Kentucky College of Medicine 12252, Lexington, Kentucky, USA; 2 Department of Molecular and Cellular Biochemistry, University of Kentucky College of Medicine 12252, Lexington, Kentucky, USA; Instituto Nacional de Tecnologia Agropecuaria, Balcarce, Buenos Aires, Argentina

**Keywords:** TmeA, invasion, N-WASP, type III secretion

## Abstract

Members of the Family *Chlamydiaceae* are obligate intracellular bacteria displaying a range of host and tissue tropisms. Genetic tractability, first established for *Chlamydia trachomatis* LGV serovar L2, is now being expanded to additional *Chlamydia* species. This capability facilitates the comparative assessment of chlamydial virulence factors during infection. All *Chlamydia* spp. express a type III secretion system, and the translocated membrane-associated protein A (TmeA) represents a pivotal effector exhibiting species-specific divergence. *Chlamydia muridarum* is a murine-specific species that has been leveraged in a host-appropriate small-animal model of disease. We utilized FRAEM mutagenesis to delete the putative *tmeA* ortholog *tc0066* in *C. muridarum* to enable pathogenesis studies. Work confirms that, similar to TmeA, TC0066 is capable of interacting with host AHNAK and N-WASP. The interaction with N-WASP was important for *C. muridarum* invasion and for subsequent intracellular development in tissue culture. During murine infections of the female genital tract, the absence of *tc0066* did not impact infection parameters, including bacterial shedding or gross pathology. These results indicate that TC0066 and *C. trachomatis* TmeA exhibit conserved functions, but these activities do not translate to detectable, overt changes to *in vivo* genital-tract pathogenesis.

**IMPORTANCE:**

*Chlamydia* spp. are prevalent pathogens capable of infecting a wide range of hosts and tissues, yet the molecular determinants mediating differences in tropism remain incompletely defined. *Chlamydia trachomatis* employs a type III secreted effector protein, TmeA, to manipulate Arp2/3-dependent actin polymerization in order to create and maintain a protected intracellular niche. The *tmeA*-encoding locus shows evidence of divergence among chlamydial species. We demonstrate herein that *C. muridarum* TC0066 represents a functionally conserved TmeA homolog and leverage this similarity to probe the contribution of this effector to chlamydial pathogenesis. Results from murine infection studies using mutant *C. muridarum* strains contrast with those from *C. trachomatis*, emphasizing the need for care when interpreting outcomes of human chlamydial infection in the murine model.

## INTRODUCTION

The genus *Chlamydia* comprises obligate intracellular bacterial pathogens that cause a wide range of important diseases in both humans and animals (1). Among these species, *Chlamydia trachomatis* is a significant human pathogen and a leading cause of sexually transmitted infections and preventable blindness worldwide. *C. trachomatis* (*Ctr*) serovars A–C are primarily associated with trachoma, an ocular infection that can lead to blindness (2), whereas serovars D–K are responsible for urogenital sexually transmitted infections in humans (3). Untreated genital infections are associated with serious reproductive health consequences, often leading to chronic inflammation and impaired fertility (4). Unlike the urogenital serovars, *C. trachomatis* serovars L1–L3 preferentially infect and replicate within regional lymphatics, causing the ulcerative sexually transmitted disease lymphogranuloma venereum (LGV) (5). *Ctr* serovar L2 has served as a common model strain for investigating the cellular microbiology of chlamydial infection (6). *Chlamydia muridarum* (*Cm*) is currently the only recognized chlamydial species that naturally infects mice. Although primary protein sequences can differ, gene synteny between *Ctr* and *Cm* is highly conserved (7). Given this degree of similarity and an ability to establish genital tract infections in mice, *Cm* has served as a valuable experimental model for studying human chlamydial pathogenesis in a small animal model (8, 9). Although significantly more divergent (7, 10), *C. pneumoniae* (*Cpn*) and *C. psittaci* (*Cps*) are two additional species mediating human respiratory and zoonotic infections, respectively (11, 12).

All *Chlamydia* spp. undergo a biphasic developmental cycle that alternates between the infectious elementary body (EB), a metabolically inactive extracellular form, and the reticulate body (RB), a metabolically active intracellular form responsible for bacterial replication (13). Following host cell entry, EBs are internalized within membrane-bound vacuoles termed inclusions, where they differentiate into RBs and undergo multiple rounds of replication. During the late stage of infection, RBs asynchronously differentiate back into EBs, which are subsequently released by host cell lysis or extrusion to initiate new rounds of infection (13). Expression of the virulence-associated type III secretion system (T3SS) is tightly coordinated with the chlamydial developmental cycle and is essential for manipulating host cellular processes throughout infection (14, 15). The EB-localized T3SS delivers effector proteins required for host cell invasion and early developmental events (16). In contrast, *de novo* effector synthesis begins during the mid-cycle stage as RBs initiate replication and supports intracellular growth and development. A subset of invasion-associated effectors, including the translocated actin-recruiting phosphoprotein (Tarp) and translocated membrane-associated effector A (TmeA), facilitate host cell entry through manipulation of the actin cytoskeleton (17). In addition, a large family of inclusion membrane proteins (Incs), which are inserted into the inclusion membrane surrounding intracellular chlamydiae, represents a major class of effectors secreted during infection (15, 18, 19).

*Ctr* TmeA is an invasion-related type III secretion effector pre-packaged within elementary bodies (EBs) to support subsequent rounds of infection. This preformed pool is secreted during entry (20), and *tmeA* mutants show reduced invasion efficiency (21). TmeA acts cooperatively with Tarp to promote actin remodeling at the entry site, with loss of either effector resulting in ~50% reduction in invasion (22, 23). Mechanistically, Tarp directly binds actin and recruits the actin-related complex (Arp2/3) to promote branched actin polymerization (24, 25). Although TmeA can interact with the transducer of Cdc42-dependent actin assembly (TACO-1) (26), TmeA-mediated actin dynamics are manifested primarily via interaction with host Neural Wiskott-Aldrich syndrome protein (N-WASP) (22, 23). Both TmeA and Tarp contribute to Dynamin 2 (Dyn2)-dependent vesicle scission during entry, yet the mechanism of TmeA-mediated Dyn2 activation remains unclear (27). TmeA also interacts with host AHNAK (20), and the function of this interaction remains undefined (21). We previously demonstrated that *de novo*–synthesized TmeA is also secreted from mature inclusions and that loss of *tmeA* impairs intracellular growth (28). In this study, TmeA-mediated N-WASP activation contributes to rerouting of host-derived material, including plasma membrane–derived endocytic cargo, toward the mature chlamydial inclusion.

The observed importance of *Ctr* TmeA in tissue culture raised the possibility of a role in overall chlamydial pathogenesis. We turned to the syntenically encoded gene product TC0066 in *C. muridarum* to test this possibility. We first confirmed conserved TmeA activities and then employed fluorescence-reported allelic exchange mutagenesis (FRAEM) (29) to generate a *tc0066* null strain. Consistent with the functions reported for TmeA in tissue culture systems, our data demonstrated that TC0066 interacts with the host proteins AHNAK and N-WASP. The interaction with N-WASP was required for efficient *C. muridarum* invasion and intracellular development. In the murine infection model, deletion of *tc0066* did not significantly affect infection parameters, including bacterial shedding or the development of gross pathology. Collectively, these findings indicate that TC0066 promotes *C. muridarum* infectivity at the cellular level, but its contribution is either dispensable or compensated for during infection *in vivo*.

## RESULTS

### TC0066 is homologous to TmeA

**Fig 1:**
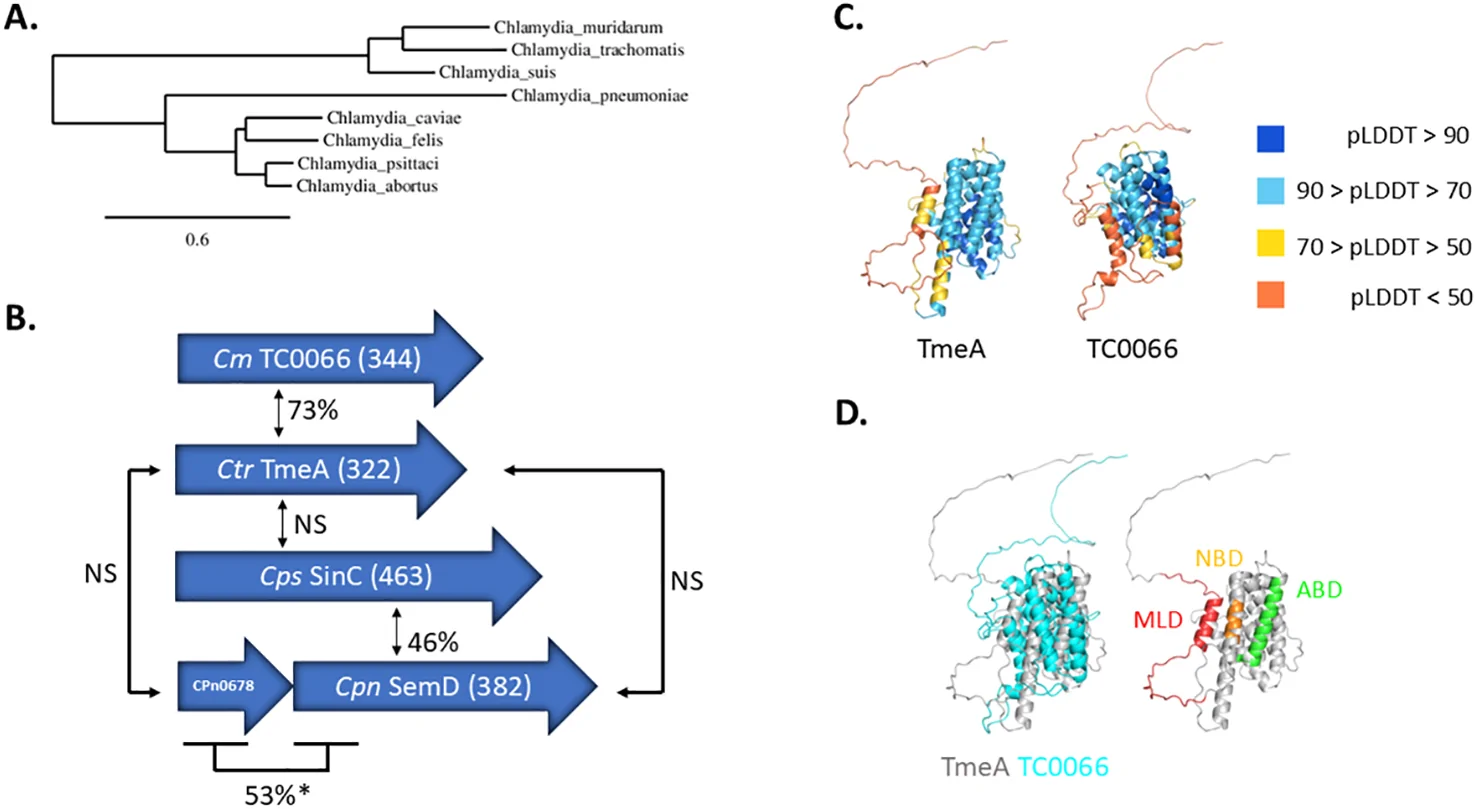
(**A**) Unrooted phylogenetic comparison generated in PhyML 3.1 (32) using neighbor-joining clustering. Input protein sequences corresponded to CTL0063 (*C. trachomatis* L2/434), TC0066 (*C. muridarum* Nigg), Cpn0677 (*C. pneumoniae* CWL029), G5Q0070 (*C. psittaci* CAL10), CAB063 (*C. abortus* S26/3), Q499_0753 (*C. suis* MD56), CF0942 (*C. felis* Fe/C-56), and CCA00062 (*C. caviae* GPIC). The scale bar corresponds to site-specific amino acid substitutions. (**B**) PSI-BLAST comparisons of indicated proteins. The total number of residues and percent similarity are indicated. NS = no apparent similarity; * = domain-confined similarity. (**C**) AlphaFold2 structural models for TmeA and TC0066 are shown in cartoon representation. The model confidence is indicated by colors corresponding to per-residue predicted local distance difference test (pLDDT) values. (**D**) Left: Superimposed structural models of TmeA (gray) and TC0066 (cyan). Right: TmeA model is shown in the same orientation, indicating the relative positions of membrane localization (MLD, red), N-WASP-binding (NBD, orange), and AHNAK-binding (ABD, green) domains.

We began addressing the possibility of functional conservation by examining the degree of similarity between *Cm* TC0066 and *Ctr* TmeA at the amino acid and structural levels. Within *C. trachomatis*, TmeA is well conserved, exhibiting 99–100% similarity via PSI-BLAST (30) among trachoma, urogenital, and LGV biovars. Importantly, the *tmeA* locus exhibits evidence of functional divergence across chlamydial species. Indeed, neighbor-clustering phylogenetic analysis using primary amino acid sequences from standard strains indicates that syntenic genes are evolutionarily divergent (31). *C. pneumoniae* and animal pathogens *C. caviae*, *felis*, *psittaci*, and *abortus* form independent clades (Fig. 1A). TC0066 is distinct yet shares a common node with *Ctr* TmeA. Both segregate to a clade containing the animal pathogen *C. suis*. Overall, the comparative distribution mirrors species-level comparisons (31). BLAST analyses indicate that TC0066 exhibits 73% overall similarity (56% identity) with TmeA (Fig. 1B). Similar searches failed to detect significant similarity of TmeA with *C. pneumoniae* SemC, SemD, or *C. psittaci* SinC. Comparative AlphaFold 2 models of TmeA and TC0066 showed similar architectures, with N-terminal disordered regions and globular α-helical C-terminal domains (Fig. 1C). Structural similarities included apparent alpha helices within the core structure containing known functional domains within TmeA (Fig. 1D). These include the membrane localization domain (MLD), spanning residues 40–80; an N-WASP-binding domain (NBD), from residues 118 to 126; and a C-terminal coiled-coil domain (residues 285–305) mediating interactions with host AHNAK (ABD). Overall, bioinformatic analyses are consistent with functional conservation between TmeA and TC0066.

### Basic TmeA functions are conserved in TC0066

**Fig 2:**
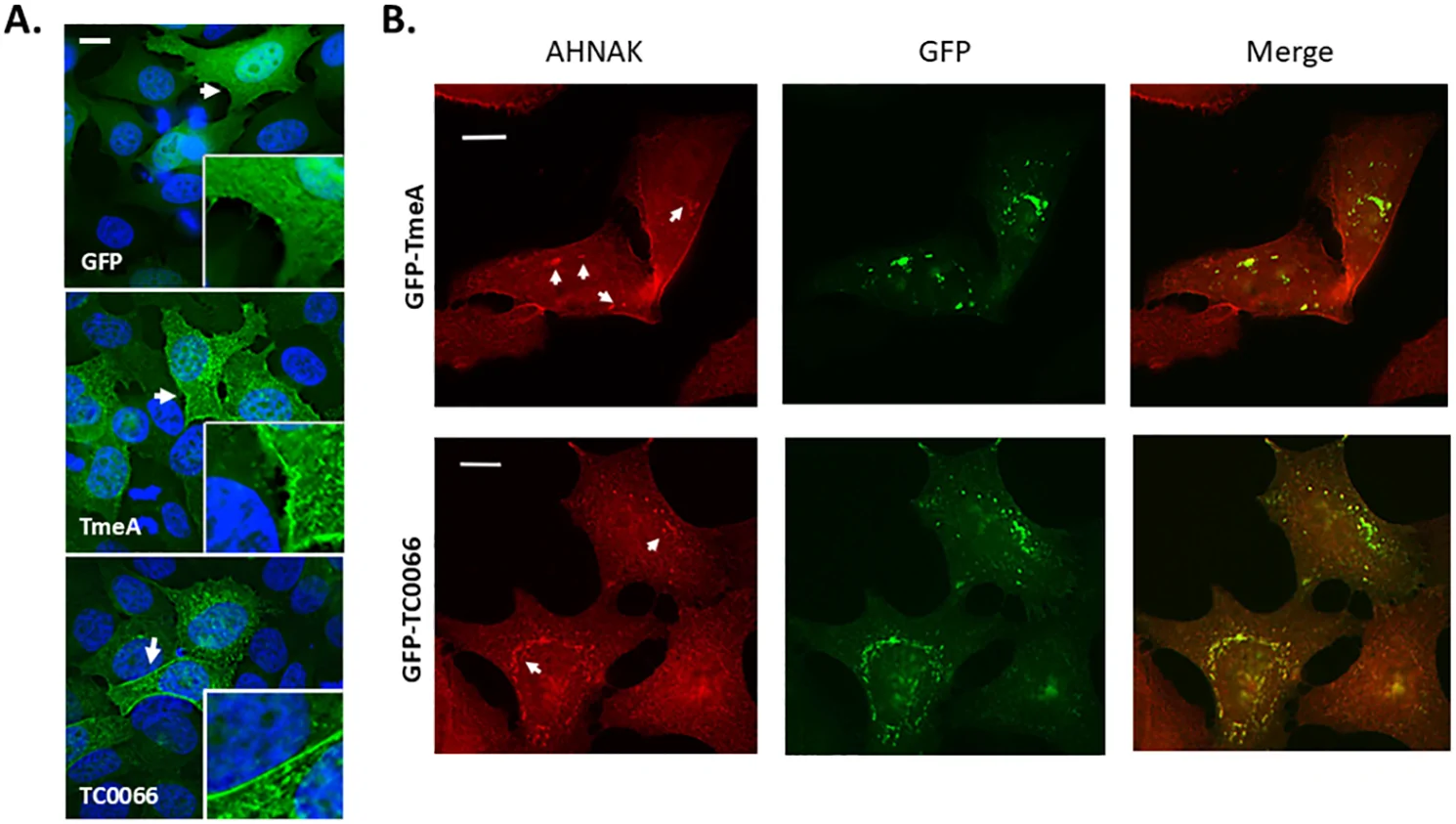
Conserved localization of GFP-tagged TC0066. HeLa cells were transfected with plasmids expressing GFP, GFP-TmeA, or GFP-TC0066. (**A**) Cultures were fixed at 24 h post-transfection and visualized for direct GFP fluorescence (green) or DAPI staining (blue). Arrows indicate areas of inset. (**B**) Transfected cultures were fixed at 30 h and stained with AHNAK-specific antibodies. AHNAK localization (red) and GFP signals (green) are depicted. Arrows indicate punctate intracellular AHNAK signal overlapping with GFP-TC0066. Bars = 10 µm.

Initial functional characterization of *Ctr* TmeA indicated an ability to peripherally associate with host membranes (33) and to interact with the host cell scaffolding protein AHNAK (20). Based on sequence and structural similarities, these capabilities should be functionally conserved for TC0066. Indeed, TC0066 has been shown to interact with AHNAK in a yeast two-hybrid system (20), similarly to TmeA. We leveraged ectopic expression of GFP-tagged TC0066 as a convenient mechanism to directly test co-localization of TmeA and AHNAK in mammalian cells (Fig. 2). HeLa cells were nucleofected with plasmid DNA encoding GFP-TmeA, GFP-TC0066, or GFP only as a negative control and cultured for 24–30 h. At 24 h, samples were fixed, counterstained with DAPI, and visualized via direct fluorescence. As previously observed (20, 28, 33), GFP-only signal remained diffuse within expressing cells, whereas GFP-TmeA concentrated at the cell periphery and in punctate, vesicle-like structures within the cytosol (Fig. 2A). GFP-TC0066 localization mirrored that of GFP-TmeA, indicating a conserved ability to associate with host membranes. During longer culture times, cytosolic GFP-TmeA accumulates in perinuclear regions (28), and these areas also contain host AHNAK (20). Thirty-hour cultures were fixed and stained with AHNAK-specific antibodies to test for co-localization (Fig. 2B). Both GFP-TmeA and GFP-TC0066 were detected in perinuclear aggregates that also stained for AHNAK. GFP-only failed to produce similar aggregates and showed no accumulated co-localization with AHNAK (Fig. S1). AHNAK signal was also concentrated at the cell periphery, consistent with typical localization to the plasma membrane. These data indicate that AHNAK is a host target of TC0066.

**Fig 3:**
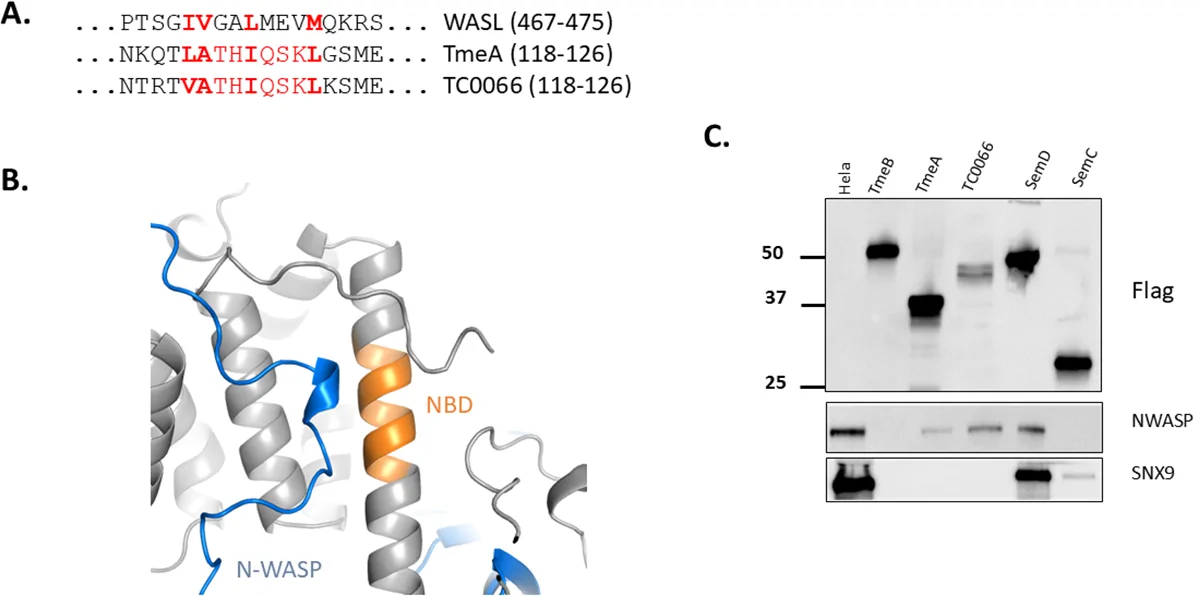
TC0066 interacts with N-WASP. (**A**) Primary amino acid sequences representing the conserved GDB ligand-binding motif from human N-WASP (WASL), *Ctr* TmeA, and *Cm* TC0066. Respective residue numbers are depicted. (**B**) A close-up view of the AlphaFold3 structural prediction depicting the interaction interface between N-WASP (blue) and TC0066 (gray), with the NBD highlighted in orange. (**C**) Whole-cell lysates were harvested from mock-treated HeLa cells (HeLa) or cells expressing the respective Flag-tagged constructs for 24 h. Immunoprecipitated material was probed on immunoblots using Flag-specific antibodies (Flag) or antibodies specific for host N-WASP or SNX9. Proteins were visualized by probing with HRP-conjugated secondary antibodies followed by chemiluminescent detection.

*Ctr* TmeA also activates host N-WASP to promote invasion (22) and mobilization of host lipids (28). TmeA contains an apparent ligand motif (23) that is thought to interact with the Cdc42/Rac interacting binding (CRIB) domain of N-WASP. The minimal domain spans TmeA residues 118–126 (23, 28) and is similar to the ligand motif of N-WASP (residues 467–475) involved in autoinhibition (Fig. 3A). This TmeA sequence is conserved in TC0066 (Fig. 3A), apart from an alternative hydrophobic valine at position 118, suggesting TC0066 can similarly interact with N-WASP. Indeed, AlphaFold3 prediction of the TC0066-N-WASP complex (Fig. 3B) reinforces this notion. Modeling predicts an interaction between the proposed NBD of TC0066 and N-WASP via a short α-helix (residues 198–205).

We probed protein-protein interactions via co-immunoprecipitation from material derived from cells ectopically expressing Flag-tagged (FT) TC0066 (Fig. 3C). HeLa cells were nucleofected with plasmids expressing N-terminally Flag-tagged TC0066 or FT-TmeA as a positive control. Cells expressing FT-SemD were included since this *Cpn* protein also binds N-WASP (34). *Ctr* TmeB and *Cpn* SemC do not interact with N-WASP (22, 35), and FT versions of each were included as negative controls. Whole-culture lysates were harvested at 24 h post-nucleofection. Flag-tagged proteins were immunoprecipitated, and recovered material was probed on immunoblots using Flag- or N-WASP-specific antibodies. Whole-cell HeLa lysates from mock nucleofections were included as a Flag antibody control and as a positive control for host protein detection. All FT proteins were detected in immunoprecipitated material. As expected, N-WASP was detected in positive control material containing FT-TmeA and FT-SemD but not in mock nucleofected, FT-TmeB, or FT-SemC negative controls. Although recovered FT-TC0066 abundance was lower than controls, N-WASP was also detected in this sample. Both SemD (34) and SemC (35) interact with sorting nexin 9 (SNX9), and SNX9 co-precipitated with both Cpn proteins. The interaction of TmeA and TC0066 with N-WASP was specific since SNX9 was not detected in either FT-TmeA or FT-TC0066-containing material. In aggregate, these data indicate that, like *Ctr* TmeA, *Cm* TC0066 has N-WASP-binding capabilities.

### Generation and phenotypic characterization of Cm Δtc0066

**Fig 4:**
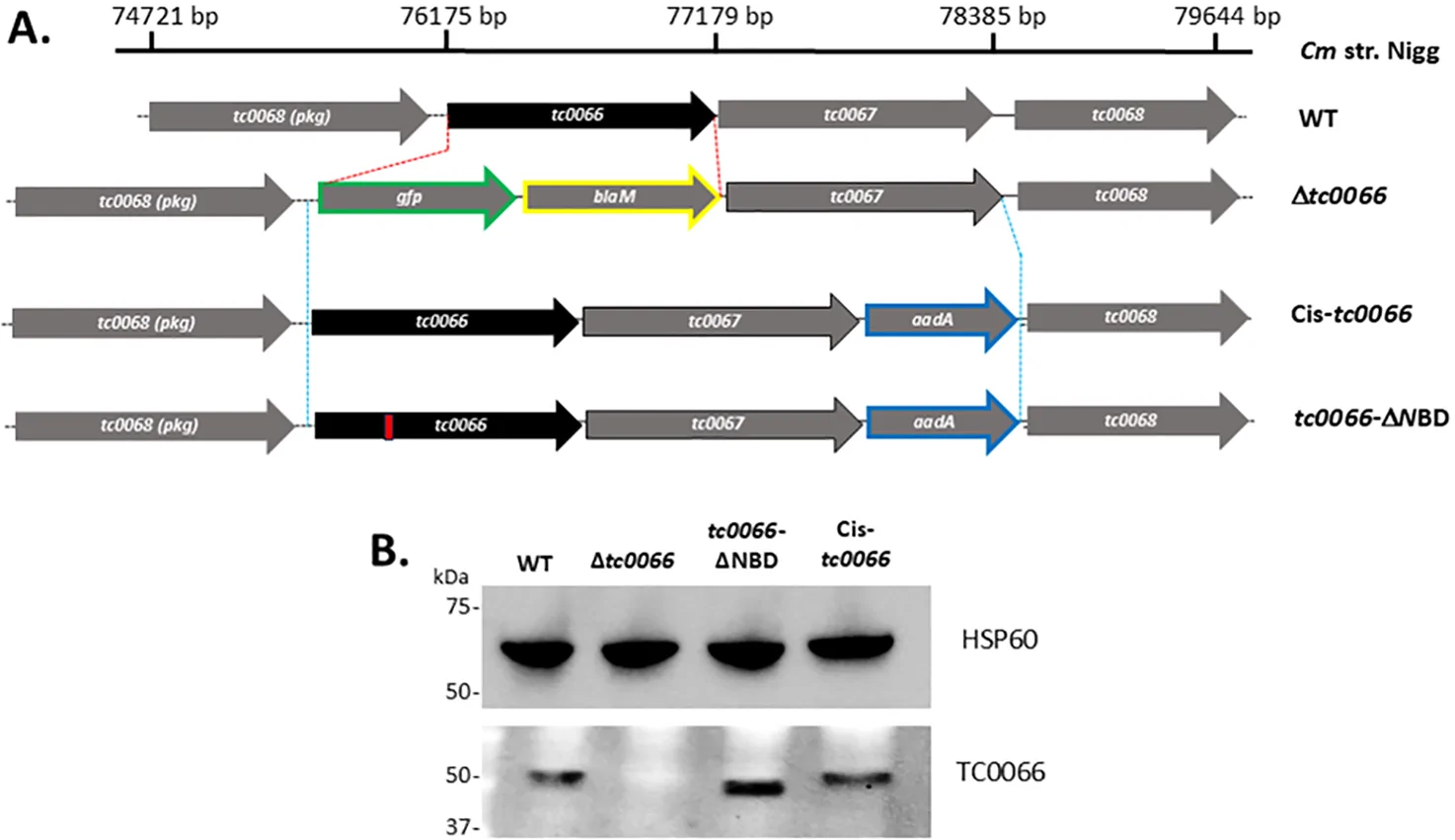
Mutagenesis of *tc0066*. (**A**) Schematic of the *tc0066* locus in *C. muridarum*. FRAEM (red lines) was used to delete the entire *tc0066* sequence and insert a cassette containing *gfp* and *blaM. Cm* Δ*tc0066* was subsequently modified by allelic replacement (blue lines) of *gfp-blaM-tc0067* with WT *tc0066* or *tc0066* lacking the putative N-WASP-binding domain (NBD; red bar). For each construct, WT *tc0067* was restored with downstream *aadA* to enable antibiotic selection. (**B**) Loss and restoration of TC0066 was confirmed by chemiluminescence immunoblot of EB material from wild-type (WT) and null (Δ*tc0066*) strains, or strains restored by cis-complementation with WT (cis-*tc0066*) or NBD-deficient (*tc0066*-ΔNBD) coding sequences. Immunoblots were probed with TC0066-specific antibodies or HSP60-specific antibodies as a loading control.

We generated a *tc0066* null strain in *Cm* to definitively test for functional conservation with *Ctr tmeA*. Fluorescence-reported allelic exchange mutagenesis (FRAEM) was used to replace *tc0066* with genes encoding both green fluorescent protein (GFP) and β-lactamase (BlaM), as previously described (29, 36, 37), to generate Δ*tc0066* (Fig. 4A). A sequential FRAEM strategy was then used, as described (38), to cis-complement Δ*tc0066* with either WT *tc0066* (cis-*tc0066*) or *tc0066* lacking codons encoding the apparent N-WASP-binding domain (*tc0066*-ΔNBD). All strains were clonally isolated via sequential limiting dilution, and whole-genome sequencing analysis confirmed anticipated manipulations of the *tc0066* locus. Polyclonal antibodies specific for His-tagged TC0066 were raised in rabbits since primary sequence divergence precluded detection by our previously generated (20) *Ctr* TmeA-specific antibodies. These antibodies were used to probe immunoblots for the presence or absence of TC0066, and loading was confirmed by probing with *Chlamydia*-specific Hsp60 antibodies (Fig. 4B). TC0066 was not detected in lysates generated from the Δ*tc0066* strain, confirming complete loss of TC0066. Complemented strains yielded similar levels of TC0066, and TC0066 lacking the NBD migrated faster than WT, as expected. These data confirmed successful generation of strains whose engineered alterations matched those of previously generated *Ctr tmeA* strains (22, 28, 29).

**Fig 5:**
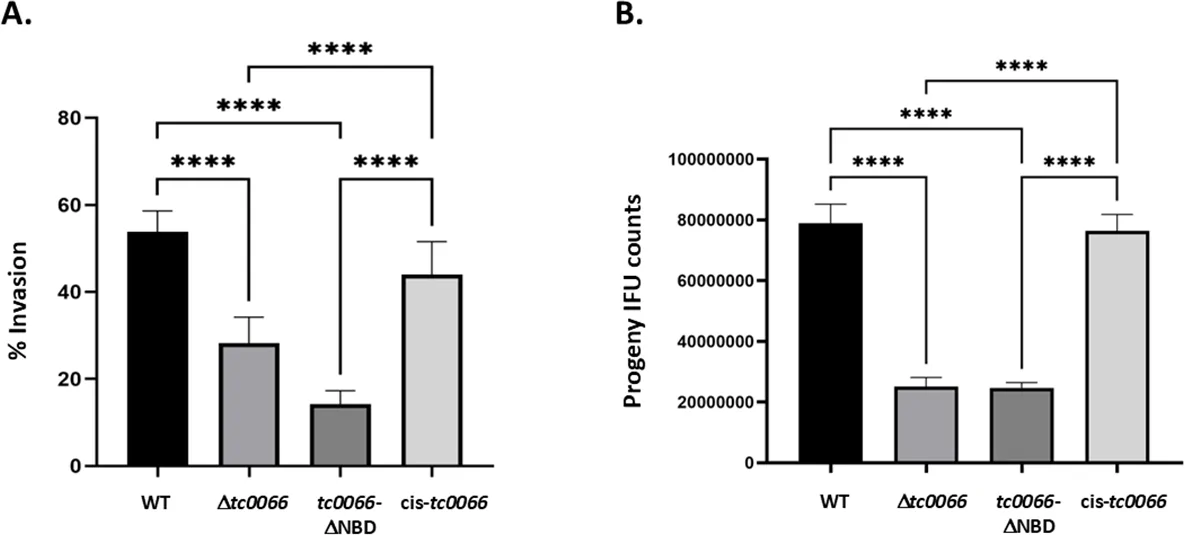
Loss of TC0066 or the N-WASP-binding domain negatively impacts chlamydial invasion and progeny production. (**A**). Invasion was assessed by infecting McCoy cells at an MOI of 10 with Cm WT, Δ*tc0066*, *tc0066*-ΔNBD, or cis-*tc0066*. Cultures were paraformaldehyde fixed after 30 min and processed for inside-out staining to assess invasion efficiency. Data are represented as percentage of internalized chlamydiae. (**B**). McCoy cells were equivalently infected with indicated strains and cultures harvested at 24 hr for enumeration of progeny EBs (IFUs/mL). All data are represented as means (bars) with standard deviations from triplicate samples of representative experiments (of three total). Statistical significance was computed using one-way ANOVA with Tukey’s multiple comparison test (****, *P* < 0.0001).

*Ctr* strains lacking TmeA, or TmeA deficient in N-WASP-binding, exhibit reduced levels of invasion (22, 23) and intracellular development (28). Comparable experiments were performed using corresponding *Cm* strains by measuring levels of invasion (Fig. 5A) and progeny EB production (Fig. 5B) in murine McCoy cells. For invasion, cells were infected with a multiplicity of infection (MO) of 10, fixed after 30 min of incubation at 37°C, and then processed for inside-out staining (39) to differentially enumerate intracellular and extracellular bacteria. WT *Cm* invasion efficiency was ca. 53% (±4.78). Loss of either TC0066 or the N-WASP-binding domain resulted in significantly decreased invasion, whereas invasion efficiency was restored in the cis-complemented *tc0066* strain. McCoy cells were equally infected (confirmed by direct counts of parallel infections to exclude invasion defects) with WT, Δ*tc0066*, *tc0066*-ΔNBD, or cis-*tc0066* and harvested at 24 h for enumeration of infectious progeny on fresh McCoy monolayers. Progeny IFUs were reduced to ca. 25% of WT for both mutant strains. Progeny counts of cis-*tc0066* were not significantly different from WT. We conclude that, like TmeA for *C. trachomatis*, the ability of TC0066 to bind host N-WASP is essential for efficient invasion and intracellular growth of *C. muridarum*. These data further support the notion of conserved function.

**Fig 6:**
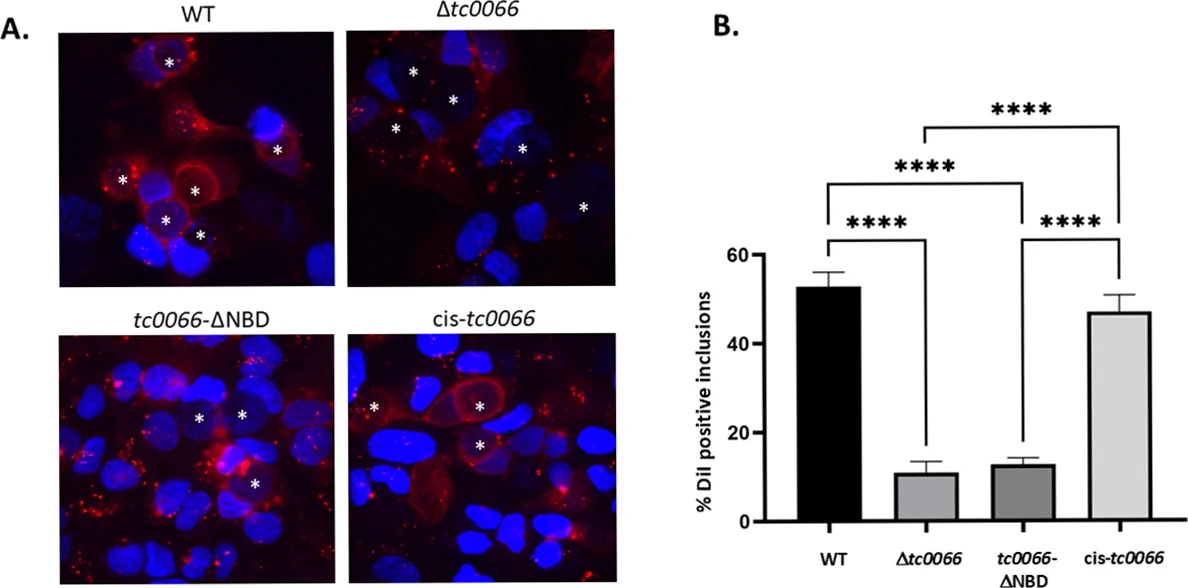
TC0066-mediated N-WASP activation is required for trafficking of lipids to the chlamydial inclusion. (**A**) Representative fluorescence images of HeLa cells infected for 24 h with wild type (WT), Δ*tc0066*, *tc0066*-ΔNBD, or cis-*tc0066* and paraformaldehyde fixed 120 min after DiI pulse. DiI (red) or DAPI (blue) signals are shown. Bar = 10 µm. Inclusions are designated by asterisk. (**B**) One hundred inclusions from triplicate cultures were scored for the presence of DiI with WT, Δ*tc0066*, *tc0066*-ΔNBD, or cis-*tc0066* inclusions and quantitation for DiI co-localization. Data are represented as means of triplicate samples with standard deviations. Statistical significance was determined by one-way ANOVA with Tukey’s multiple comparison test (****, *P* < 0.0001).

Decreased intracellular replication in the absence of *tc0066* raised the possibility that TC0066 supports the lipid trafficking observed for TmeA. The lipophilic tracer DiI accumulates at the inclusion membrane in *C. trachomatis*-infected cells, and the N-WASP-binding domain of TmeA is an important mediator of this process (28). We utilized HeLa cells to test for a similar *Cm* requirement since DiI signal is spatially resolved better in these flattened cells. Cultures were probed at 24 h post-infection with DiI for 120 min, counterstained with DAPI, and visualized via epifluorescence (Fig. 6A). DiI signal was detected in association with WT and cis-*tc0066* inclusions but not those from Δ*tc0066* or *tc0066*-ΔNBD infected cells (Fig. 6A). Quantification of inclusions clearly surrounded by concentrated DiI signal (Fig. 6B) indicated that DiI was trafficked to 53% (±3.3%) or 47% (±3.7%) of WT or cis-*tc0066* inclusions, respectively. Loss of *tc0066* or the N-WASP-binding domain limited trafficking to 11% (±2.5%) or 13% (±1.4%) of inclusions, respectively. These data indicate that TC0066 also supports trafficking of lipids from the host plasma membrane to the chlamydial inclusion. Overall, our analyses indicate functional conservation between *Ctr* TmeA and *Cm* TC0066.

### Cm TC0066 is dispensable in the murine infection model

**Fig 7:**
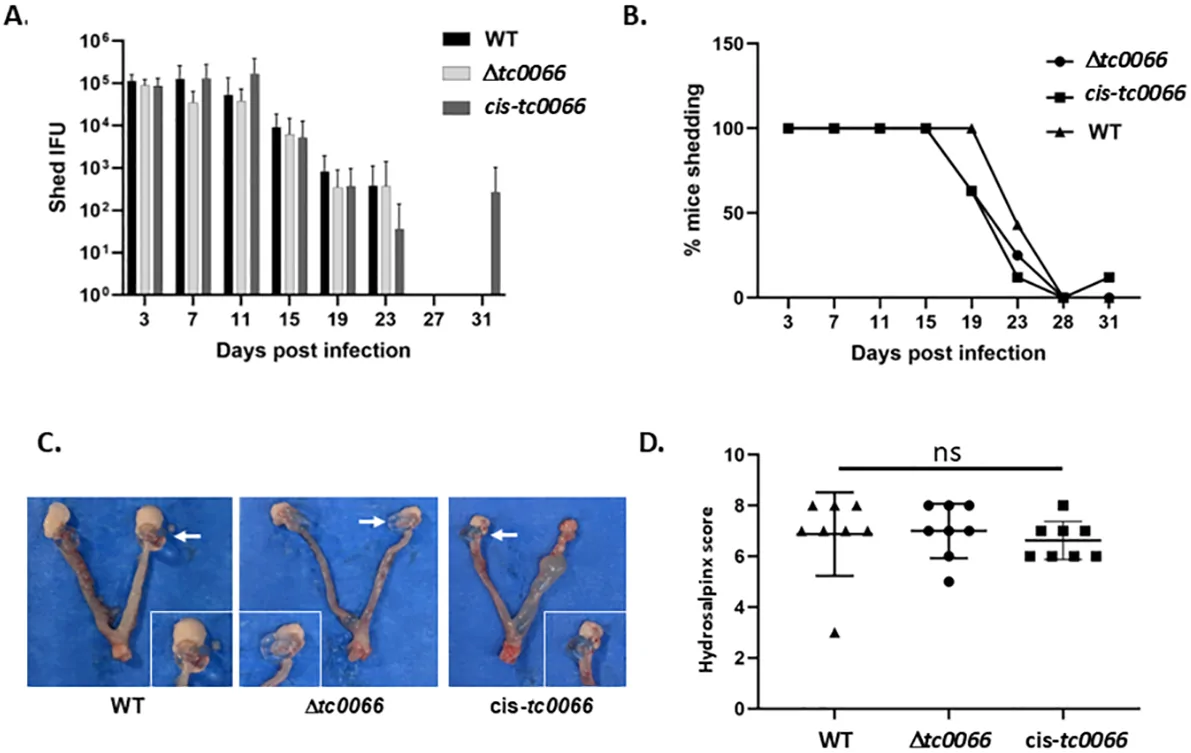
TC0066 is dispensable for overt pathogenesis in the murine infection model. Groups of four mice were challenged intravaginally with equal infectious units of WT, Δ*tc0066*, or cis-*tc0066 Cm*, and shed infectious particles were enumerated from days 3 to 31. All data are pooled from two independent experiments. (**A**) Average progeny numbers shed and (**B**) percentage of mice actively shedding chlamydiae at different days post-infection. No statistically significant differences were detected by a two-way ANOVA test. Reproductive tracts were excised on day 54 post-infection and assessed for gross pathology. (**C**) Representative images are shown from each group and areas of hydrosalpinx (white arrows) are highlighted in insets. (**D**) Hydrosalpinx severity score was determined and data are plotted for each mouse. A Kruskal-Wallis with Dunn’s multiple comparison was performed to address significance (ns = not significant).

We proceeded to test potential contributions of TC0066 in pathogenesis given that loss of TC0066 attenuated infection in tissue culture. We utilized the well-established murine infection model to directly assess genital tract pathogenesis with a host-appropriate pathogen. Groups of four female C57BL/6 mice were challenged cervico-vaginally with 10^5^ IFU of WT, Δ*tc0066*, or cis-*tc0066*, and shed infectious particles were enumerated from vaginal swabs taken every 4 days. Data were pooled from two independent experiments. We did not detect any significant differences in shed IFU (Fig. 7A) or percentage of mice actively shedding chlamydiae (Fig. 7B). Mice were euthanized on day 55 to evaluate gross pathology in upper genital tracts (UGTs). All strains supported visual indications of oviduct hydrosalpinx (Fig. 7C). Neither the incidence nor severity of hydrosalpinx (Fig. 7D) showed significant differences across strains. In aggregate, these data indicate that TC0066 is not required for *Cm* growth or induction of the overt pathology associated with chlamydial infection in mice.

## DISCUSSION

Members of the *Chlamydiaceae* occupy a highly conserved intracellular niche within epithelial cells where they manifest a common bi-phasic developmental cycle. All *Chlamydiaceae* employ a virulence-associated type III secretion system (40) to deploy effector proteins that create and maintain the privileged intracellular niche (15). Despite this general conservation, species display a wide range of host and tissue tropism (31). The bulk of this diversity is postulated to be derived from proteins encoded within the chlamydial plasticity zone (41), where variability in gene content has been correlated with species-specific differences in infection biology (31). Variability in effector content could also contribute to species-specific biology. For example, the panel of host-interactive inclusion membrane proteins (Incs) varies among species (19, 42), with the increased overall number of putative Incs in *C. pneumoniae* possibly conferring a broader host range (19). Moreover, syntenically encoded effectors often show divergence at the primary sequence level (31), raising an additional possibility for species-specific functions.

The locus encoding *Ctr* TmeA has garnered considerable interest due to apparent divergence across species. TmeA is a ca. 34 kDa T3S effector protein secreted during invasion (20) and later during intracellular development (28), where it associates with host membranes via a discrete membrane localization domain (33). TmeA has a high degree of sequence identity via BLASTP across all *C. trachomatis* biovars. However, similar searches of syntenically encoded proteins, including the comparatively larger SinC of *C. psittaci*, failed to reveal significant similarity. Interestingly, the *Cpn* locus uniquely contains two genes (designated *semC* and *semD*) encoding apparent paralogs of TmeA. Evidence supports divergent overall function for both SinC and SemD. *Cps* SinC localizes to the inner nuclear membrane and may influence transport in that compartment (43). Insertional inactivation of the *C. caviae* ortholog does not impact growth in HeLa cells but does decrease lethality in a chicken embryo infection model (44). *Ccav* SinC does not mediate lipid trafficking, and *C. caviae* inclusions do not accumulate the lipophilic dye DiI as does *C. trachomatis* (28). Lack of tractable mutagenesis in *C. pneumoniae* has precluded definitive functional characterization of SemD. However, cell biology data implicate SemD in entry. SemD associates with host membranes to induce curvature, recruits G-actin, and interacts with the BAR-domain-containing protein sorting nexin 9 (34). Interestingly, SemD interacts with and activates N-WASP independently of Cdc42, similarly to TmeA (45). This observation implicates some degree of functional conservation despite sequence divergence. *C. muridarum* is an additional model species used prominently to model chlamydial pathogenesis in mice (9). We therefore sought to test whether *Cm* TC0066 is a TmeA ortholog in order to leverage the murine model to explore potential contributions of this effector to pathogenesis. Phylogenetically, *C. muridarum* is more closely related to *C. trachomatis* than to other animal pathogens or *C. pneumoniae* (41). Bioinformatic analyses indicated that *Cm* TC0066 is a TmeA ortholog. Primary sequence similarity is robust at 73%. Structurally, predicted secondary and tertiary folding of TC0066 mirrors that of TmeA. Subsequent functional evidence provided herein supports the conclusion that TC0066 exerts similar activities in *C. muridarum*.

*Ctr* TmeA engages multiple host proteins via distinct domains during invasion and intracellular development. Loss of *tmeA* negatively impacts invasion efficiency (21–23) and subsequent Golgi-independent trafficking of host lipids to mature inclusions (28). TmeA interferes with AHNAK-mediated actin bundling activity, but the precise role of this activity during infection remains undefined and does not appear to impact chlamydial entry or growth (21). Co-localization of ectopically expressed TC0066 with AHNAK confirmed our previous work (20) and the conservation of this interaction in *C. muridarum*. Additionally, *Ctr* TmeA promotes efficient dynamin 2-mediated vesicle fission during entry, yet this activity does not manifest as overt defects in invasion (27). The mechanism of dynamin 2 activation remains unknown, but pharmacologic inhibition of dynamin 2 interferes with TmeA-dependent lipid trafficking during intracellular development (28).

According to current working models, *Ctr* TmeA-mediated manipulation of host actin dynamics represents this effector’s most prominent role during infection, and this activity is dependent on engagement of host N-WASP. We therefore focused our efforts on testing whether TC0066 functioned similarly in this regard. N-WASP induces Arp2/3-mediated branched polymerization (46) and is associated with endocytic cargo internalization, Golgi-ER transport, and vesicle rocketing (47). Activation of N-WASP typically requires the active RhoGTPase Cdc42 (48). TmeA interaction with the N-WASP CRIB domain (23) bypasses this requirement, and TmeA is sufficient to induce N-WASP-dependent actin polymerization *in vitro* (22). Deletion of the TmeA N-WASP domain phenocopies complete loss of *tmeA* during invasion, growth, and lipid trafficking (28). Culmination of these effects is likely conserved in *C. muridarum* since loss of *tc0066* decreased both invasion and intracellular replication. These effects were N-WASP dependent since the *tc0066*-ΔNBD strain yielded similar results.

These results contrasted with outcomes previously obtained characterizing *tarp* null strains (49). Loss of either *Ctr* TmeA or *Ctr* Tarp manifests as decreased invasion that is further decreased in a corresponding double mutant (22, 23). These effectors appear to contribute equally, yet distinctly, during *C. trachomatis* entry. Deletion of *Cm tarp* interferes with invasion efficiency (50), but not intracellular growth (49). Interestingly, loss of Tarp decreases intracellular growth of *C. trachomatis* serovar D but not L2 or *C. muridarum* (49). Tarp is a multi-domain, multifunctional effector impacting actin polymerization and host cell signaling pathways (51). Tarp exhibits a more significant degree of variation even among *C. trachomatis* biovars, and alterations correlate with clinical manifestations (52). Although Tarp in *C. trachomatis* and *C. muridarum* share 59% overall sequence identity, *Cm* Tarp lacks the tyrosine-containing tandem repeat domains targeted for phosphorylation by host kinases (53). These differences could explain alternate infection biology. Regardless, the observation that *Cm* TC0066 is required for *C. muridarum* invasion suggests that this effector may have a more prominent role in entry. This is perhaps not terribly surprising given the alternative requirements noted for *in vitro* infections (54).

Intravaginal infection with murine-specific *C. muridarum* has historically proven to be a robust small animal model to characterize key events during chlamydial infection in a host-appropriate format (9). Critical pathogenesis (55) parameters such as chlamydial infectivity, ascension into the UGT, and subsequent ability to induce pathology can be readily modeled. The *tc0066* null strain did not exhibit attenuated infection in the mouse model. Mice shed infectious particles at levels comparable to WT. Gross pathology was also similar to WT, indicating that there was no defect in ascension into the UGT. This was somewhat surprising given our past studies with *tarp*-deficient *C. muridarum*. The *Cm* Δ*tarp* strain exhibits a ca. 100-fold decrease in shedding at day 3 compared to WT, is cleared from mice more rapidly, and induces decreased levels of hydrosalpinx (49). These data indicated that Tarp is important for establishing UGT infection *in vivo*. It was, therefore, curious that the *Cm tc0066* strain failed to show a similar phenotype since Δ*tc0066* phenocopied Δ*tmeA* for decreased invasion and intracellular growth in tissue culture.

The results were also somewhat surprising given that *Ctr* Δ*tmeA* is cleared more rapidly than WT after intravaginal infection of mice (21). One caveat to those studies is that human *Chlamydia* species are confined to the lower genital tract when introduced intravaginally in the mouse model, where they primarily infect vaginal epithelial cells (56) instead of typical columnar epithelial cells of the cervix (55). Unlike *C. muridarum*, innate immunity is sufficient to clear *C. trachomatis* infections (57). These data raise the possibility that TmeA is important in the lower genital tract but dispensable during cervical and UGT infection. Alternatively, redundant functions may mask the importance of *Cm* TC0066 in mice. *C. muridarum* may express mechanisms that are absent in *C. trachomatis*. In addition, loss of *Cm* TC0066 does not completely abolish lipid trafficking, raising the possibility of compensatory mechanisms. Future work will address these possibilities.

The expanding genetic tractability of *Chlamydia* species is enabling a more refined view of infection biology, with an emphasis on species-level comparisons. Overall, our analyses support the assignment of TC0066 as *Cm* TmeA and reinforce a model where TmeA-mediated alterations in actin polymerization contribute to infection at multiple levels. Our mouse data underscore the need for caution when applying *C. trachomatis* (especially LGV strains) to the murine infection model. In aggregate, the data also indicate that attenuation in tissue culture does not ensure an *in vivo* phenotype, and vice versa. Future work will explore these interesting differences to investigate species-specific infection biology in *Chlamydia*.

## MATERIALS AND METHODS

### Cell culture and organisms

Studies employed *C. muridarum* strain Nigg as the parent wild-type strain. Chlamydiae were routinely propagated in murine fibroblast McCoy cells (CRL-1696; ATCC) for genetic manipulations. Transformations were accomplished as described (36, 38) using either 400 µg/mL spectinomycin or 0.6 ng/mL penicillin G for selection. Experiments were performed where indicated in McCoy or human Hela 299 epithelial cells (CCL-1.2; ATCC). Unless otherwise noted, McCoy and Hela cells were grown in RPMI 160 medium containing 2 mM l-glutamine (Life Technologies) supplemented with 10% (vol/vol) heat-inactivated fetal bovine serum (Thermo Fisher). Infections were established by centrifugation of EBs onto cell monolayers at 900 × *g* for 1 h at 20°C or 15 min at 4°C when appropriate for invasion assays. All cultures were routinely maintained at 37°C in an environment with 5% CO_2_ and 95% humidified air.

*C. muridarum* ΔTC0066 was generated from clonal WT *C. muridarum*, via FRAEM as described (29). Allelic replacement was used to delete the entire *tc0066* sequence and insert a cassette containing *gfp* and *blaM*. The FRAEM plasmid pKW-bla-∆TC0066-CM*ori* was generated by sequential insertion of ~3 kb 5′ and 3′ arm sequences (representing sequences upstream and downstream of the *tc0066* gene), amplified from *C. muridarum* genomic DNA. Cis-complemented strains were subsequently generated from ΔTC0066 by transforming with pKW-CMori-cis-*tc0066* or pKW-CMori-*tc0066*-ΔNBD to generate cis-complemented strains expressing WT or TC0066-ΔNBD, respectively. pKW-CMori-cis-*tc0066* containing WT *tc0066*, *aadA*, 5′, and 3′ homology arms was synthesized (GenScript). Deletion of codons 118–126, representing the NBD, was accomplished by divergent PCR to yield pKW-CMori-*tc0066*-ΔNBD. All cloning primers were custom DNA oligonucleotides (Integrated DNA Technologies) and are listed in Table S1. All plasmids were sequence verified. Isolation of clonal populations was accomplished for all *Chlamydia* strains by limiting dilution as described (36), and manipulations were confirmed via whole-genome sequencing (SeqCenter) of genomic DNA.

### Immunodetection and microscopy

For immunoblot analyses, proteins were separated on 4–15% SDS-PAGE gels (Bio-Rad) and transferred to 0.45 µm PVDF membranes (Millipore). Commercially available primary antibodies specific for HSP60 (A57-B9) (Santa Cruz), Flag-M2 (Sigma), NWASP, SNX9, or GAPDH (Cell Signaling) were used. Custom antibodies were used to detect AHNAK (20) and TC0066. For anti-TC0066, full-length *tc0066* was synthesized and inserted in-frame into pET-15b (GenScript Biotech, Corp) to generate His-tagged (HT) TC0066. HT-TC0066 was purified from NEB10ß lysates and used to hyperimmunize New Zealand White rabbits (ThermoFisher Custom Services) to generate TC0066-specific antibodies. All proteins were detected using peroxidase-conjugated secondary antibodies (Sigma) and visualized with Amersham ECL Plus reagents (GE Healthcare UK Limited). For fluorescence imaging, cells were fixed with 4% paraformaldehyde for 10 min at room temperature and subsequently washed with phosphate-buffered saline (PBS). GFP-TmeA and GFP-TC0066-expressing cells, as well as cells labeled with 1,1′-dioctadecyl-3,3,3′,3′-tetra methylindocarbocyanine perchlorate (DiI; Invitrogen), were visualized by direct fluorescence. Chlamydiae were routinely detected by DAPI staining of nucleic acids. Samples were examined using a Nikon E800 Eclipse epifluorescence microscopy equipped with a 100× oil immersion objective. All images were processed identically using the auto contrast and unsharp mask filter functions in Adobe Photoshop 6.0 (Adobe Systems).

### Co-localization studies

Vybrant DiI Cell-Labeling Solution (Invitrogen) was used to monitor lipid trafficking from the plasma membrane as described (28). Briefly, HeLa cells grown on 12-mm glass coverslips were infected with *Chlamydia* by centrifugation. At 24 hpi, cells were incubated with 5 µM DiI for 5 min on ice, washed three times with PBS, and subsequently incubated within RPMI/FBS for 30 min at 37°C. Cells were fixed with 4% paraformaldehyde and stained with DAPI. Coverslips were mounted using ProLong Diamond Antifade Mountant (Invitrogen) and analyzed by fluorescence microscopy. Inclusions were scored as DiI-positive when a continuous rim-like fluorescence signal was observed around the entire inclusion membrane. Data are presented as the percentage of DiI-positive inclusions.

### Fitness assays

For the invasion assays, McCoy cells were seeded into 24-well plates containing 12 mm coverslips. Density gradient purified EBs of *C. muridarum* were used at a multiplicity of infection (MOI) of 10. Infections were initiated by centrifugation at 900 × *g* for 15 min at 4°C, followed by incubation at 37°C for 30 min. Cultures were fixed with 4% paraformaldehyde at room temperature for 15 min then rinsed with phosphate-buffered saline (PBS). The extracellular EBs were labeled using murine chlamydial lipopolysaccharide (LPS)–specific antibody followed by secondary antibody conjugated to Alexa-488 (anti-mouse). Cells were then permeabilized with 0.01% Triton X-100 and stained with rabbit L2-specific antibody followed by secondary antibody conjugated to Alexa-594 (anti-rabbit). Percent invasion was determined by enumerating intracellular and extracellular chlamydiae in 10 fields of view. EB internalization was calculated using the following formula: $\frac{\left(total\;EBs-external\;EBs\right)}{total\;red\;EBs}\;\times \;100=\%\;invasion$. These experiments were performed three times.

For progeny assays, infected cells were mechanically disrupted at 24 h post-infection. The released progeny EBs were used to infect fresh McCoy monolayers by centrifugation at 900 × *g* for 1 h, followed by an incubation at 37°C for 24 h. To determine the IFUs, cultures were fixed with methanol and stained with Hsp60-specific primary antibodies, followed by Alexa-594-conjugated anti-mouse secondary antibodies. In parallel, primary cultures were methanol-fixed to assess the number of inclusions, allowing normalization of progeny output to the level of the initial infection. An equivalent number of infectious particles were used for the initial infection of each strain, and inclusion counts were determined as described above. All growth data were generated from triplicate replicates for each strain and condition, with all experiments repeated at least three times independently.

### Murine genital tract infection

All mouse procedures were performed in accordance with protocols approved by the University of Kentucky IACUC (protocol #2019-3222). Female mice C57BL/6 were purchased from the Jackson Laboratory (USA), housed at the DLAR facility, and provided food and water *ad libitum*. Intravaginal infection was performed as has been previously described (58). Briefly, groups of four female mice aged 6–8 weeks were treated subcutaneously with 2.5 mg depo-medroxyprogesterone acetate (Pfizer) 5 days prior to intravaginal infection with 10^5^ IFU of density gradient purified EBs of *C. muridarum* WT, Δ*tc0066*, and cis-*tc0066*. Shed bacteria were enumerated from vaginal swabs beginning on day 3 then every 4 days until day 31 by determination of infectious forming units in HeLa cells. At day 55, gross pathology was assessed via necropsy. Each uterine horn was examined individually for hydrosalpinx as described (59). Individual horns were scored from 1 to 4, and cumulative scores were generated for each mouse for a range of 0–8. Scores are defined as described (59), with 0 assigned for no visible hydrosalpinx. Severity scores were assigned based on hydrosalpinx visible only after amplification (score = 1), clearly visible to the naked eye but smaller than the ovary (score = 2), similar in size to the ovary (score = 3), or larger than the ovary (score = 4).

### Structural modeling

The AlphaFold2 models of TmeA and TC0066 were retrieved from the AlphaFold database (60). The complex between TC0066 and N-WASP was modeled using AlphaFold3 in standard regime (61). The structural comparisons were performed using Dali (62). The structural figures were prepared using PyMOL (The PyMOL Molecular Graphics System, Version 3.0 Schrödinger, LLC).

### Statistics

Statistical analyses were performed using GraphPad Prism 9 version 9.5.1 (GraphPad Software Inc.), as indicated in the appropriate figure legends, and statistical significance was defined as *P* < 0.05. Unless otherwise noted, the presented data are representative results from experiments repeated at least three times. For animal studies, two independent experiments were performed for each analysis, with four mice per group per treatment.

## Data Availability

Whole-genome sequence data can be accessed at BioProject-NCBI under BioProject PRJNA1514026. All additional data required to assess conclusions are present in the paper or the supplemental material.

## SUPPLEMENTAL MATERIAL

Table S1 and Fig. S1 spectrum.02359-26-s0001.docx Cloning primers and GFP localization.

ASM does not own the copyrights to Supplemental Material that may be linked to, or accessed through, an article. The authors have granted ASM a non-exclusive, world-wide license to publish the Supplemental Material files. Please contact the corresponding author directly for reuse.
