# Supplementary material for: Chlamydia muridarum TC0066 is required for invasion and intracellular development in tissue culture but dispensable in the murine infection model
Source: Microbiol Spectr. 2026 Sep 30;14(11):e02359-26. doi: 10.1128/spectrum.02359-26 (PMC13729174; doi:10.1128/spectrum.02359-26)
Supplement: Supplementary file 1 [file spectrum.02359-26-s0001.docx]

**Table S1. Cloning Primers**

| Name | Sequence (5’-3’)^a^ | Function |
| --- | --- | --- |
| TC0066-Sal1-S | GAACTCAAAATTTTACCACAGAAGTGGTTAGATCGACTGTT  ATCCAAAAGTACAGATTATGGACG | Insert 5’sequences flanking *tc0066* into pKW-CM*ori* |
| TC0066-Sal1-AS | GTATGCTATACGAAGTAGGGTCGGATCCTCCGTCAAC AATAACTTTTAATCA ACTGTTTTATTA AATTTTAAA AAC | Insert 3’sequences flanking *tc0066* into pKW-CM*ori* |
| TC0066-Sbf1-S | CATTATACGAGTTATGACCTGCGGGGAAGAAGTAGTGAAT AGT GTA GGT CCT ATT G | Insert 3’sequences flanking *tc0066* into pKW-CM*ori* |
| TC0066-sbf1-AS | GTTCTACGGGGTCTGACGGTTCTTGTCCTTCTTGAGA  GAAAATGACACATCC | Insert 3’sequences flanking *tc0066* into pKW-CM*ori* |
| 118-126aa-divPCR-S | TTAAAATCCATGGAGAAACAGTCATC | Deletion of NBD from pKW-CMCori-cis-*tc0066* |
| 118-126aa-divPCR-AS | CCGTGTATTCTGTGCACG | Deletion of NBD from pKW-CMCori-cis-*tc0066* |

^a^ Primer sequences are listed in the 5’→3’ direction

**Figure S1**. HeLa cells were nucleofected with a plasmid expressing GFP, fixed at 30 hrs, and stained with AHNAK-specific antibodies. AHNAK localization (red), GFP signals (green), and merged images are depicted. Bar = 10 µm.
